# Supplementary material for: Unravelling the Impact of Cyclic Mechanical Stretch in Keratoconus—A Transcriptomic Profiling Study
Source: Int J Mol Sci. 2023 Apr 18;24(8):7437. doi: 10.3390/ijms24087437 (PMC10139219; doi:10.3390/ijms24087437)
Supplement: Supplementary file 1 [file ijms-24-07437-s001.zip › Supplementary Table S10.pdf]

Supplementary Table S10. Molecular function gene ontology terms for 199 KC-altered genes responsive to TGFβ1 treatment (FDR ≤ 0.05).

	Homo sapiens (REF)	Client Text Box Input (▼ Hierarchy NEW! ?)					
GO molecular function complete	#	#	expected	Fold Enrichment	+/-	raw P value	FDR
extracellular matrix structural constituent	174	13	1.66	7.81	+	2.84E-08	1.41E-04
protein binding	14448	169	138.24	1.22	+	5.86E-07	9.71E-04
↳binding	16594	186	158.77	1.17	+	4.99E-08	1.24E-04
Unclassified	2297	5	21.98	.23	-	1.70E-05	2.11E-02
