# Supplementary material for: Unravelling the Impact of Cyclic Mechanical Stretch in Keratoconus—A Transcriptomic Profiling Study
Source: Int J Mol Sci. 2023 Apr 18;24(8):7437. doi: 10.3390/ijms24087437 (PMC10139219; doi:10.3390/ijms24087437)
Supplement: Supplementary file 1 [file ijms-24-07437-s001.zip › Supplementary Table S11.pdf]

Supplementary Table S11. Cellular component gene ontology terms for KC-altered genes responsive to TGFβ1 treatment (FDR ≤ 0.05).

	Homo sapiens (REF)	Client Text Box Input (Hierarchy NEW!)					
GO cellular component complete	#	#	expected	Fold Enrichment	+/-	raw P value	FDR
basement membrane	101	9	.97	9.31	+	1.05E-06	3.05E-04
↳collagen-containing extracellular matrix	434	24	4.15	5.78	+	1.23E-11	8.37E-09
↳extracellular matrix	575	31	5.50	5.63	+	1.75E-14	3.58E-11
↳external encapsulating structure	576	31	5.51	5.62	+	1.83E-14	1.87E-11
↳cell periphery	6460	102	61.81	1.65	+	4.36E-09	1.78E-06
anchored component of membrane	171	9	1.64	5.50	+	5.69E-05	1.45E-02
extracellular space	3430	65	32.82	1.98	+	2.52E-08	8.57E-06
↳extracellular region	4396	82	42.06	1.95	+	2.38E-10	1.21E-07
