# Supplementary material for: Unravelling the Impact of Cyclic Mechanical Stretch in Keratoconus—A Transcriptomic Profiling Study
Source: Int J Mol Sci. 2023 Apr 18;24(8):7437. doi: 10.3390/ijms24087437 (PMC10139219; doi:10.3390/ijms24087437)
Supplement: Supplementary file 1 [file ijms-24-07437-s001.zip › Supplementary Table S12.pdf]

Supplementary Table S12. Reactome pathways for KC-altered genes responsive to TGFβ1 treatment (FDR ≤ 0.05).

PANTHER Reactome pathways

	Homo sapiens (REF)	Client Text Box Input (Hierarchy NEW!)					
Reactome pathways	#	#	expected	Fold Enrichment	+/-	raw P value	FDR
Interferon alpha/beta signaling	68	9	.65	13.83	+	4.75E-08	1.19E-04
↳Interferon Signaling	197	11	1.88	5.84	+	4.99E-06	6.22E-03
↳Cytokine Signaling in Immune system	697	20	6.67	3.00	+	1.68E-05	1.40E-02

DAVID Reactome pathways

Sublist	Category	Term	RT	Genes	Count	%	P-Value	FDR	Fisher Exact
	REACTOME_PATHWAY	Interferon alpha/beta signaling	RT		10	5.1	4.9E-8	2.5E-5	3.3E-9
	REACTOME_PATHWAY	Interferon Signaling	RT		11	5.6	4.2E-5	1.1E-2	7.5E-6
	REACTOME_PATHWAY	Cytokine Signaling in Immune system	RT		20	10.3	1.5E-4	2.6E-2	5.6E-5
	REACTOME_PATHWAY	Extracellular matrix organization	RT		12	6.2	2.7E-4	3.5E-2	6.8E-5
