# Supplementary material for: Unravelling the Impact of Cyclic Mechanical Stretch in Keratoconus—A Transcriptomic Profiling Study
Source: Int J Mol Sci. 2023 Apr 18;24(8):7437. doi: 10.3390/ijms24087437 (PMC10139219; doi:10.3390/ijms24087437)
Supplement: Supplementary file 1 [file ijms-24-07437-s001.zip › Supplementary Table S3.pdf]

**Supplementary Table S3.** Biological process gene ontology terms for 479 DEGs from HKCs (TGFβ1 + CMS) vs. HCFs (TGFβ1 + CMS) (FDR ≤ 0.05).

| | Homo sapiens (REF) | Client Text Box Input ( Hierarchy NEW! ) | | | | | |
|---|---|---|---|---|---|---|---|
| GO biological process complete | # | # | expected | Fold Enrichment | +/- | raw P value | FDR |
| MDA-5 signaling pathway | 4 | 3 | .09 | 34.55 | + | 3.13E-04 | 2.74E-02 |
| ↳cytoplasmic pattern recognition receptor signaling pathway in response to virus | 11 | 4 | .24 | 16.75 | + | 2.28E-04 | 2.18E-02 |
| ↳defense response to virus | 253 | 27 | 5.49 | 4.92 | + | 6.73E-11 | 8.79E-08 |
| ↳response to virus | 357 | 33 | 7.75 | 4.26 | + | 1.74E-11 | 4.54E-08 |
| ↳response to other organism | 1429 | 62 | 31.02 | 2.00 | + | 3.38E-07 | 1.06E-04 |
| ↳biological process involved in interspecies interaction between organisms | 1602 | 65 | 34.78 | 1.87 | + | 2.00E-06 | 4.89E-04 |
| ↳response to external biotic stimulus | 1432 | 63 | 31.09 | 2.03 | + | 1.91E-07 | 6.50E-05 |
| ↳response to biotic stimulus | 1478 | 63 | 32.09 | 1.96 | + | 5.05E-07 | 1.47E-04 |
| ↳response to stimulus | 8209 | 247 | 178.22 | 1.39 | + | 9.18E-11 | 1.03E-07 |
| ↳response to external stimulus | 2469 | 107 | 53.60 | 2.00 | + | 4.98E-12 | 3.91E-08 |
| ↳defense response to symbiont | 254 | 27 | 5.51 | 4.90 | + | 7.30E-11 | 8.80E-08 |
| ↳defense response to other organism | 1068 | 49 | 23.19 | 2.11 | + | 1.70E-06 | 4.29E-04 |
| ↳defense response | 1478 | 68 | 32.09 | 2.12 | + | 1.14E-08 | 5.44E-06 |
| ↳response to stress | 3466 | 107 | 75.25 | 1.42 | + | 1.62E-04 | 1.68E-02 |
| ↳signal transduction | 4887 | 161 | 106.10 | 1.52 | + | 8.88E-09 | 4.35E-06 |

| | | | | | | | |
|---|---|---|---|---|---|---|---|
| ↳regulation of cellular process | 11187 | 301 | 242.88 | 1.24 | + | 3.76E-08 | 1.51E-05 |
| ↳regulation of biological process | 11806 | 320 | 256.32 | 1.25 | + | 8.56E-10 | 5.84E-07 |
| ↳biological regulation | 12544 | 329 | 272.34 | 1.21 | + | 3.30E-08 | 1.36E-05 |
| ↳signaling | 5231 | 178 | 113.57 | 1.57 | + | 4.96E-11 | 7.06E-08 |
| ↳cellular process | 15044 | 368 | 326.61 | 1.13 | + | 6.74E-06 | 1.39E-03 |
| ↳cell communication | 5342 | 178 | 115.98 | 1.53 | + | 2.91E-10 | 2.68E-07 |
| ↳cellular response to stimulus | 6569 | 206 | 142.62 | 1.44 | + | 6.49E-10 | 4.62E-07 |
| ↳regulation of response to stimulus | 4034 | 142 | 87.58 | 1.62 | + | 2.09E-09 | 1.22E-06 |
| ↳intracellular signal transduction | 1511 | 58 | 32.80 | 1.77 | + | 3.56E-05 | 5.26E-03 |
| interleukin-27-mediated signaling pathway | 7 | 4 | .15 | 26.32 | + | 5.90E-05 | 7.52E-03 |
| ↳cell surface receptor signaling pathway | 2174 | 77 | 47.20 | 1.63 | + | 2.58E-05 | 4.08E-03 |
| ↳cellular response to cytokine stimulus | 714 | 34 | 15.50 | 2.19 | + | 4.13E-05 | 5.74E-03 |
| ↳cellular response to organic substance | 2026 | 70 | 43.99 | 1.59 | + | 1.58E-04 | 1.65E-02 |
| ↳cellular response to chemical stimulus | 2616 | 97 | 56.79 | 1.71 | + | 2.12E-07 | 6.94E-05 |
| ↳response to chemical | 4060 | 124 | 88.15 | 1.41 | + | 5.60E-05 | 7.20E-03 |
| ↳response to cytokine | 808 | 39 | 17.54 | 2.22 | + | 7.21E-06 | 1.47E-03 |
| protein mono-ADP-ribosylation | 12 | 5 | .26 | 19.19 | + | 2.12E-05 | 3.50E-03 |
| sequestering of extracellular ligand from receptor | 10 | 4 | .22 | 18.42 | + | 1.70E-04 | 1.74E-02 |

| | | | | | | | |
|---|---|---|---|---|---|---|---|
| ↳extracellular negative regulation of signal transduction | 12 | 4 | .26 | 15.35 | + | 2.99E-04 | 2.68E-02 |
| ↳extracellular regulation of signal transduction | 12 | 4 | .26 | 15.35 | + | 2.99E-04 | 2.66E-02 |
| ↳regulation of signal transduction | 2989 | 112 | 64.89 | 1.73 | + | 7.10E-09 | 3.84E-06 |
| ↳regulation of cell communication | 3369 | 125 | 73.14 | 1.71 | + | 1.09E-09 | 6.81E-07 |
| ↳regulation of signaling | 3381 | 127 | 73.40 | 1.73 | + | 3.33E-10 | 2.90E-07 |
| ↳negative regulation of signal transduction | 1257 | 57 | 27.29 | 2.09 | + | 2.88E-07 | 9.21E-05 |
| ↳negative regulation of cell communication | 1357 | 59 | 29.46 | 2.00 | + | 7.45E-07 | 2.05E-04 |
| ↳negative regulation of cellular process | 4732 | 155 | 102.73 | 1.51 | + | 3.22E-08 | 1.36E-05 |
| ↳negative regulation of biological process | 5314 | 180 | 115.37 | 1.56 | + | 4.44E-11 | 6.96E-08 |
| ↳negative regulation of signaling | 1363 | 60 | 29.59 | 2.03 | + | 4.70E-07 | 1.39E-04 |
| ↳negative regulation of response to stimulus | 1620 | 70 | 35.17 | 1.99 | + | 7.99E-08 | 2.98E-05 |
| positive regulation of non-canonical Wnt signaling pathway | 15 | 4 | .33 | 12.28 | + | 6.05E-04 | 4.52E-02 |
| ↳regulation of Wnt signaling pathway | 332 | 18 | 7.21 | 2.50 | + | 5.43E-04 | 4.13E-02 |
| ↳positive regulation of signal transduction | 1528 | 59 | 33.17 | 1.78 | + | 2.59E-05 | 4.06E-03 |
| ↳positive regulation of signaling | 1701 | 65 | 36.93 | 1.76 | + | 1.33E-05 | 2.40E-03 |
| ↳positive regulation of biological process | 6304 | 200 | 136.86 | 1.46 | + | 5.71E-10 | 4.26E-07 |
| ↳positive regulation of cell communication | 1696 | 65 | 36.82 | 1.77 | + | 1.28E-05 | 2.34E-03 |
| ↳positive regulation of cellular process | 5670 | 175 | 123.10 | 1.42 | + | 1.49E-07 | 5.18E-05 |

| | | | | | | | |
|---|---|---|---|---|---|---|---|
| ↳positive regulation of response to stimulus | 2223 | 79 | 48.26 | 1.64 | + | 1.59E-05 | 2.77E-03 |
| fibroblast migration | 19 | 5 | .41 | 12.12 | + | 1.29E-04 | 1.44E-02 |
| ↳cell migration | 903 | 41 | 19.60 | 2.09 | + | 1.45E-05 | 2.55E-03 |
| ↳cell motility | 1061 | 44 | 23.03 | 1.91 | + | 6.09E-05 | 7.70E-03 |
| mesenchymal cell proliferation | 28 | 5 | .61 | 8.23 | + | 6.15E-04 | 4.57E-02 |
| ↳cell population proliferation | 718 | 31 | 15.59 | 1.99 | + | 4.14E-04 | 3.33E-02 |
| negative regulation of viral genome replication | 57 | 10 | 1.24 | 8.08 | + | 1.43E-06 | 3.67E-04 |
| ↳regulation of viral genome replication | 87 | 10 | 1.89 | 5.29 | + | 4.10E-05 | 5.80E-03 |
| ↳regulation of viral life cycle | 142 | 12 | 3.08 | 3.89 | + | 1.15E-04 | 1.31E-02 |
| ↳regulation of viral process | 164 | 14 | 3.56 | 3.93 | + | 2.86E-05 | 4.36E-03 |
| ↳negative regulation of viral process | 95 | 14 | 2.06 | 6.79 | + | 7.97E-08 | 3.05E-05 |
| cell adhesion mediated by integrin | 41 | 6 | .89 | 6.74 | + | 4.57E-04 | 3.62E-02 |
| ↳cell adhesion | 969 | 57 | 21.04 | 2.71 | + | 3.14E-11 | 6.16E-08 |
| positive regulation of interferon-beta production | 41 | 6 | .89 | 6.74 | + | 4.57E-04 | 3.60E-02 |
| ↳regulation of cytokine production | 742 | 37 | 16.11 | 2.30 | + | 5.52E-06 | 1.22E-03 |
| ↳regulation of multicellular organismal process | 2750 | 109 | 59.70 | 1.83 | + | 5.29E-10 | 4.15E-07 |
| ↳positive regulation of cytokine production | 481 | 24 | 10.44 | 2.30 | + | 2.43E-04 | 2.27E-02 |
| ↳positive regulation of multicellular organismal process | 1515 | 63 | 32.89 | 1.92 | + | 1.13E-06 | 2.94E-04 |
| ↳regulation of interferon-beta production | 58 | 7 | 1.26 | 5.56 | + | 4.49E-04 | 3.59E-02 |
| cellular response to type I interferon | 44 | 6 | .96 | 6.28 | + | 6.41E-04 | 4.72E-02 |
| ↳response to type I interferon | 52 | 8 | 1.13 | 7.09 | + | 3.80E-05 | 5.46E-03 |

| | | | | | | | |
|---|---|---|---|---|---|---|---|
| ↳innate immune response | 832 | 43 | 18.06 | 2.38 | + | 4.12E-07 | 1.24E-04 |
| ↳immune response | 1621 | 64 | 35.19 | 1.82 | + | 5.52E-06 | 1.20E-03 |
| ↳immune system process | 2429 | 90 | 52.74 | 1.71 | + | 6.34E-07 | 1.81E-04 |
| negative regulation of innate immune response | 76 | 10 | 1.65 | 6.06 | + | 1.42E-05 | 2.53E-03 |
| ↳negative regulation of response to external stimulus | 391 | 21 | 8.49 | 2.47 | + | 3.49E-04 | 2.91E-02 |
| ↳regulation of response to external stimulus | 973 | 45 | 21.12 | 2.13 | + | 3.39E-06 | 7.93E-04 |
| ↳negative regulation of response to biotic stimulus | 114 | 10 | 2.48 | 4.04 | + | 3.21E-04 | 2.78E-02 |
| ↳regulation of response to biotic stimulus | 364 | 22 | 7.90 | 2.78 | + | 3.02E-05 | 4.55E-03 |
| ↳regulation of defense response | 642 | 33 | 13.94 | 2.37 | + | 9.17E-06 | 1.77E-03 |
| ↳negative regulation of defense response | 236 | 15 | 5.12 | 2.93 | + | 3.31E-04 | 2.81E-02 |
| regulation of actin filament depolymerization | 56 | 7 | 1.22 | 5.76 | + | 3.70E-04 | 3.07E-02 |
| ↳regulation of cellular component organization | 2364 | 82 | 51.32 | 1.60 | + | 2.63E-05 | 4.04E-03 |
| ↳regulation of actin polymerization or depolymerization | 158 | 12 | 3.43 | 3.50 | + | 2.91E-04 | 2.62E-02 |
| ↳regulation of actin filament length | 161 | 12 | 3.50 | 3.43 | + | 3.41E-04 | 2.86E-02 |
| ↳regulation of actin cytoskeleton organization | 364 | 21 | 7.90 | 2.66 | + | 8.53E-05 | 1.01E-02 |
| ↳regulation of actin filament-based process | 405 | 24 | 8.79 | 2.73 | + | 1.79E-05 | 3.06E-03 |
| ↳regulation of cellular component size | 372 | 21 | 8.08 | 2.60 | + | 1.14E-04 | 1.30E-02 |
| ↳cellular component organization | 5523 | 161 | 119.91 | 1.34 | + | 2.54E-05 | 4.06E-03 |
| ↳cellular component organization or biogenesis | 5727 | 161 | 124.34 | 1.29 | + | 1.88E-04 | 1.89E-02 |

| | | | | | | | |
|---|---|---|---|---|---|---|---|
| ↳regulation of anatomical structure size | 511 | 27 | 11.09 | 2.43 | + | 4.53E-05 | 6.08E-03 |
| ↳regulation of actin filament organization | 276 | 16 | 5.99 | 2.67 | + | 5.53E-04 | 4.19E-02 |
| regulation of establishment of planar polarity | 58 | 7 | 1.26 | 5.56 | + | 4.49E-04 | 3.57E-02 |
| ↳regulation of anatomical structure morphogenesis | 924 | 50 | 20.06 | 2.49 | + | 8.44E-09 | 4.27E-06 |
| ↳regulation of developmental process | 2489 | 103 | 54.04 | 1.91 | + | 2.11E-10 | 2.07E-07 |
| collagen fibril organization | 60 | 7 | 1.30 | 5.37 | + | 5.41E-04 | 4.14E-02 |
| ↳supramolecular fiber organization | 580 | 29 | 12.59 | 2.30 | + | 7.47E-05 | 9.15E-03 |
| ↳extracellular matrix organization | 280 | 30 | 6.08 | 4.94 | + | 5.22E-12 | 2.73E-08 |
| ↳extracellular structure organization | 281 | 30 | 6.10 | 4.92 | + | 5.67E-12 | 2.22E-08 |
| ↳external encapsulating structure organization | 283 | 30 | 6.14 | 4.88 | + | 6.67E-12 | 2.09E-08 |
| nephron tubule development | 83 | 9 | 1.80 | 4.99 | + | 1.49E-04 | 1.59E-02 |
| ↳renal tubule development | 87 | 10 | 1.89 | 5.29 | + | 4.10E-05 | 5.75E-03 |
| ↳tube development | 885 | 39 | 19.21 | 2.03 | + | 5.37E-05 | 6.96E-03 |
| ↳anatomical structure development | 5144 | 176 | 111.68 | 1.58 | + | 3.94E-11 | 6.87E-08 |
| ↳developmental process | 5677 | 190 | 123.25 | 1.54 | + | 2.51E-11 | 5.62E-08 |
| ↳multicellular organism development | 4228 | 156 | 91.79 | 1.70 | + | 3.35E-12 | 5.25E-08 |
| ↳multicellular organismal process | 6581 | 202 | 142.88 | 1.41 | + | 8.33E-09 | 4.35E-06 |
| ↳tissue development | 1726 | 72 | 37.47 | 1.92 | + | 1.91E-07 | 6.36E-05 |
| ↳renal system development | 308 | 21 | 6.69 | 3.14 | + | 8.49E-06 | 1.66E-03 |

| | | | | | | | |
|---|---|---|---|---|---|---|---|
| ↳system development | 3838 | 141 | 83.33 | 1.69 | + | 9.63E-11 | 1.01E-07 |
| ↳urogenital system development | 350 | 24 | 7.60 | 3.16 | + | 1.77E-06 | 4.40E-04 |
| ↳nephron epithelium development | 106 | 10 | 2.30 | 4.35 | + | 1.86E-04 | 1.88E-02 |
| ↳kidney epithelium development | 137 | 11 | 2.97 | 3.70 | + | 3.32E-04 | 2.80E-02 |
| ↳kidney development | 299 | 21 | 6.49 | 3.24 | + | 5.55E-06 | 1.19E-03 |
| ↳animal organ development | 3254 | 116 | 70.65 | 1.64 | + | 6.91E-08 | 2.71E-05 |
| ↳nephron development | 135 | 12 | 2.93 | 4.09 | + | 7.38E-05 | 9.11E-03 |
| negative regulation of cytokine-mediated signaling pathway | 75 | 8 | 1.63 | 4.91 | + | 3.83E-04 | 3.16E-02 |
| ↳negative regulation of response to cytokine stimulus | 79 | 8 | 1.72 | 4.66 | + | 5.26E-04 | 4.11E-02 |
| transport across blood-brain barrier | 86 | 9 | 1.87 | 4.82 | + | 1.91E-04 | 1.89E-02 |
| ↳vascular transport | 86 | 9 | 1.87 | 4.82 | + | 1.91E-04 | 1.90E-02 |
| ↳vascular process in circulatory system | 267 | 17 | 5.80 | 2.93 | + | 1.33E-04 | 1.45E-02 |
| ↳circulatory system process | 498 | 24 | 10.81 | 2.22 | + | 5.14E-04 | 4.03E-02 |
| inner ear morphogenesis | 109 | 11 | 2.37 | 4.65 | + | 5.12E-05 | 6.75E-03 |
| ↳ear morphogenesis | 130 | 13 | 2.82 | 4.61 | + | 1.18E-05 | 2.19E-03 |
| ↳ear development | 232 | 17 | 5.04 | 3.38 | + | 2.61E-05 | 4.05E-03 |
| ↳sensory organ development | 582 | 32 | 12.64 | 2.53 | + | 4.80E-06 | 1.07E-03 |
| ↳sensory organ morphogenesis | 279 | 23 | 6.06 | 3.80 | + | 1.48E-07 | 5.28E-05 |
| ↳animal organ morphogenesis | 1003 | 46 | 21.78 | 2.11 | + | 4.29E-06 | 9.75E-04 |
| ↳anatomical structure morphogenesis | 2237 | 94 | 48.57 | 1.94 | + | 9.69E-10 | 6.33E-07 |

| | | | | | | | |
|---|---|---|---|---|---|---|---|
| ↳embryonic organ morphogenesis | 301 | 18 | 6.53 | 2.75 | + | 1.78E-04 | 1.81E-02 |
| ↳embryonic organ development | 454 | 24 | 9.86 | 2.43 | + | 1.30E-04 | 1.43E-02 |
| ↳embryo development | 1059 | 45 | 22.99 | 1.96 | + | 3.48E-05 | 5.20E-03 |
| ↳embryonic morphogenesis | 591 | 32 | 12.83 | 2.49 | + | 5.67E-06 | 1.20E-03 |
| ↳inner ear development | 204 | 14 | 4.43 | 3.16 | + | 2.51E-04 | 2.32E-02 |
| integrin-mediated signaling pathway | 101 | 10 | 2.19 | 4.56 | + | 1.29E-04 | 1.44E-02 |
| response to retinoic acid | 113 | 10 | 2.45 | 4.08 | + | 3.00E-04 | 2.66E-02 |
| actomyosin structure organization | 114 | 10 | 2.48 | 4.04 | + | 3.21E-04 | 2.79E-02 |
| ↳actin cytoskeleton organization | 549 | 30 | 11.92 | 2.52 | + | 1.08E-05 | 2.06E-03 |
| ↳actin filament-based process | 611 | 33 | 13.27 | 2.49 | + | 4.07E-06 | 9.38E-04 |
| eye morphogenesis | 160 | 12 | 3.47 | 3.45 | + | 3.24E-04 | 2.79E-02 |
| homophilic cell adhesion via plasma membrane adhesion molecules | 170 | 12 | 3.69 | 3.25 | + | 5.39E-04 | 4.17E-02 |
| ↳cell-cell adhesion | 543 | 34 | 11.79 | 2.88 | + | 9.55E-08 | 3.48E-05 |
| mesenchymal cell differentiation | 171 | 12 | 3.71 | 3.23 | + | 5.66E-04 | 4.27E-02 |
| ↳cell differentiation | 3519 | 119 | 76.40 | 1.56 | + | 6.82E-07 | 1.91E-04 |
| ↳cellular developmental process | 3542 | 119 | 76.90 | 1.55 | + | 1.01E-06 | 2.69E-04 |
| ↳mesenchyme development | 239 | 17 | 5.19 | 3.28 | + | 3.70E-05 | 5.38E-03 |
| negative regulation of cell migration | 288 | 17 | 6.25 | 2.72 | + | 3.08E-04 | 2.72E-02 |
| ↳negative regulation of cell motility | 303 | 19 | 6.58 | 2.89 | + | 6.59E-05 | 8.20E-03 |
| ↳negative regulation of locomotion | 339 | 22 | 7.36 | 2.99 | + | 1.08E-05 | 2.04E-03 |

| | | | | | | | |
|---|---|---|---|---|---|---|---|
| ↳regulation of locomotion | 1034 | 55 | 22.45 | 2.45 | + | 3.30E-09 | 1.85E-06 |
| ↳regulation of cell motility | 989 | 54 | 21.47 | 2.51 | + | 1.50E-09 | 9.06E-07 |
| ↳regulation of cell migration | 929 | 53 | 20.17 | 2.63 | + | 4.69E-10 | 3.87E-07 |
| positive regulation of nervous system development | 279 | 16 | 6.06 | 2.64 | + | 6.18E-04 | 4.57E-02 |
| ↳positive regulation of developmental process | 1339 | 53 | 29.07 | 1.82 | + | 4.33E-05 | 5.95E-03 |
| ↳regulation of nervous system development | 452 | 25 | 9.81 | 2.55 | + | 3.56E-05 | 5.22E-03 |
| ↳regulation of multicellular organismal development | 1383 | 58 | 30.03 | 1.93 | + | 2.60E-06 | 6.27E-04 |
| regulation of cell morphogenesis | 314 | 18 | 6.82 | 2.64 | + | 2.90E-04 | 2.63E-02 |
| regulation of ERK1 and ERK2 cascade | 303 | 17 | 6.58 | 2.58 | + | 5.34E-04 | 4.14E-02 |
| cell morphogenesis involved in neuron differentiation | 440 | 23 | 9.55 | 2.41 | + | 2.04E-04 | 1.99E-02 |
| ↳cell development | 1725 | 60 | 37.45 | 1.60 | + | 3.98E-04 | 3.22E-02 |
| ↳cell morphogenesis | 713 | 34 | 15.48 | 2.20 | + | 4.08E-05 | 5.82E-03 |
| ↳neuron development | 843 | 37 | 18.30 | 2.02 | + | 9.96E-05 | 1.15E-02 |
| ↳neuron differentiation | 1065 | 41 | 23.12 | 1.77 | + | 5.40E-04 | 4.15E-02 |
| ↳generation of neurons | 1134 | 45 | 24.62 | 1.83 | + | 1.53E-04 | 1.61E-02 |
| ↳neurogenesis | 1290 | 51 | 28.01 | 1.82 | + | 5.13E-05 | 6.71E-03 |
| ↳nervous system development | 2191 | 88 | 47.57 | 1.85 | + | 3.10E-08 | 1.35E-05 |
| muscle structure development | 499 | 26 | 10.83 | 2.40 | + | 7.52E-05 | 9.14E-03 |
| regulation of neuron projection development | 445 | 23 | 9.66 | 2.38 | + | 2.27E-04 | 2.18E-02 |
| ↳regulation of plasma membrane bounded cell projection organization | 640 | 30 | 13.89 | 2.16 | + | 1.68E-04 | 1.74E-02 |
| ↳regulation of cell projection organization | 656 | 30 | 14.24 | 2.11 | + | 2.10E-04 | 2.03E-02 |

| | | | | | | | |
|---|---|---|---|---|---|---|---|
| blood vessel development | 510 | 26 | 11.07 | 2.35 | + | 9.86E-05 | 1.14E-02 |
| ↳vasculature development | 532 | 27 | 11.55 | 2.34 | + | 7.62E-05 | 9.12E-03 |
| ↳circulatory system development | 909 | 42 | 19.73 | 2.13 | + | 8.19E-06 | 1.62E-03 |
| neuron projection morphogenesis | 479 | 24 | 10.40 | 2.31 | + | 2.31E-04 | 2.18E-02 |
| ↳plasma membrane bounded cell projection morphogenesis | 484 | 24 | 10.51 | 2.28 | + | 2.62E-04 | 2.39E-02 |
| ↳cell projection morphogenesis | 489 | 25 | 10.62 | 2.35 | + | 1.31E-04 | 1.44E-02 |
| ↳cell part morphogenesis | 508 | 25 | 11.03 | 2.27 | + | 3.24E-04 | 2.77E-02 |
| ↳cellular component morphogenesis | 604 | 31 | 13.11 | 2.36 | + | 1.79E-05 | 3.09E-03 |
| positive regulation of cell migration | 532 | 26 | 11.55 | 2.25 | + | 2.42E-04 | 2.27E-02 |
| ↳positive regulation of cell motility | 558 | 26 | 12.11 | 2.15 | + | 3.87E-04 | 3.16E-02 |
| ↳positive regulation of locomotion | 574 | 27 | 12.46 | 2.17 | + | 2.61E-04 | 2.39E-02 |
| negative regulation of multicellular organismal process | 1039 | 50 | 22.56 | 2.22 | + | 3.67E-07 | 1.13E-04 |
| tissue morphogenesis | 568 | 27 | 12.33 | 2.19 | + | 2.31E-04 | 2.19E-02 |
| tube morphogenesis | 673 | 31 | 14.61 | 2.12 | + | 1.45E-04 | 1.56E-02 |
| anatomical structure formation involved in morphogenesis | 941 | 43 | 20.43 | 2.10 | + | 7.45E-06 | 1.50E-03 |
| negative regulation of cell population proliferation | 713 | 31 | 15.48 | 2.00 | + | 3.85E-04 | 3.16E-02 |
| ↳regulation of cell population proliferation | 1674 | 68 | 36.34 | 1.87 | + | 8.97E-07 | 2.43E-04 |
| positive regulation of cell differentiation | 884 | 38 | 19.19 | 1.98 | + | 9.61E-05 | 1.12E-02 |
| ↳regulation of cell differentiation | 1581 | 64 | 34.32 | 1.86 | + | 2.70E-06 | 6.41E-04 |
| central nervous system development | 1034 | 43 | 22.45 | 1.92 | + | 7.54E-05 | 9.10E-03 |
| cell-cell signaling | 1083 | 43 | 23.51 | 1.83 | + | 2.56E-04 | 2.36E-02 |

| | | | | | | | |
|---|---|---|---|---|---|---|---|
| regulation of apoptotic process | 1468 | 54 | 31.87 | 1.69 | + | 2.00E-04 | 1.98E-02 |
| ↳regulation of programmed cell death | 1498 | 54 | 32.52 | 1.66 | + | 3.30E-04 | 2.81E-02 |
| regulation of localization | 2104 | 70 | 45.68 | 1.53 | + | 3.89E-04 | 3.16E-02 |
| regulation of molecular function | 3094 | 102 | 67.17 | 1.52 | + | 1.80E-05 | 3.04E-03 |
| cell cycle | 1249 | 11 | 27.12 | .41 | - | 5.72E-04 | 4.29E-02 |
| Unclassified | 2725 | 22 | 59.16 | .37 | - | 1.41E-08 | 6.33E-06 |
| RNA processing | 868 | 4 | 18.84 | .21 | - | 6.26E-05 | 7.85E-03 |
| ↳gene expression | 2314 | 22 | 50.24 | .44 | - | 5.95E-06 | 1.24E-03 |
| ↳RNA metabolic process | 1635 | 15 | 35.50 | .42 | - | 1.17E-04 | 1.32E-02 |
| ↳nucleic acid metabolic process | 2276 | 23 | 49.41 | .47 | - | 2.19E-05 | 3.57E-03 |
| ↳nucleobase-containing compound metabolic process | 2825 | 34 | 61.33 | .55 | - | 8.65E-05 | 1.02E-02 |
| ↳organic cyclic compound metabolic process | 3292 | 41 | 71.47 | .57 | - | 4.51E-05 | 6.09E-03 |
| ↳heterocycle metabolic process | 2999 | 35 | 65.11 | .54 | - | 2.22E-05 | 3.59E-03 |
| ↳cellular aromatic compound metabolic process | 3050 | 37 | 66.22 | .56 | - | 4.66E-05 | 6.20E-03 |
| ↳cellular nitrogen compound metabolic process | 3573 | 44 | 77.57 | .57 | - | 1.13E-05 | 2.11E-03 |
| ncRNA metabolic process | 536 | 1 | 11.64 | .09 | - | 2.02E-04 | 1.98E-02 |
| detection of chemical stimulus involved in sensory perception of smell | 443 | 0 | 9.62 | < 0.01 | - | 1.50E-04 | 1.59E-02 |
| ↳detection of chemical stimulus involved in sensory perception | 489 | 0 | 10.62 | < 0.01 | - | 4.37E-05 | 5.96E-03 |
| ↳detection of chemical stimulus | 526 | 0 | 11.42 | < 0.01 | - | 1.99E-05 | 3.32E-03 |
| ↳sensory perception of chemical stimulus | 551 | 1 | 11.96 | .08 | - | 1.38E-04 | 1.49E-02 |
