# Supplementary material for: Unravelling the Impact of Cyclic Mechanical Stretch in Keratoconus—A Transcriptomic Profiling Study
Source: Int J Mol Sci. 2023 Apr 18;24(8):7437. doi: 10.3390/ijms24087437 (PMC10139219; doi:10.3390/ijms24087437)
Supplement: Supplementary file 1 [file ijms-24-07437-s001.zip › Supplementary Table S4.pdf]

Supplementary Table S4. Molecular function gene ontology terms for 479 DEGs from HKCs (TGFβ1 + CMS) vs. HCFs (TGFβ1 + CMS) (FDR ≤ 0.05).

	Homo sapiens (REF)	Client Text Box Input (Hierarchy NEW!)					
GO molecular function complete	#	#	expected	Fold Enrichment	+/-	raw P value	FDR
TAP binding	8	4	.17	23.03	+	8.70E-05	3.33E-02
↳protein binding	14448	367	313.68	1.17	+	1.27E-08	2.10E-05
↳binding	16594	412	360.27	1.14	+	2.05E-11	1.02E-07
extracellular matrix binding	56	8	1.22	6.58	+	6.10E-05	2.53E-02
extracellular matrix structural constituent	174	21	3.78	5.56	+	1.24E-09	3.08E-06
integrin binding	159	14	3.45	4.06	+	2.08E-05	1.15E-02
↳signaling receptor binding	1602	69	34.78	1.98	+	1.13E-07	9.38E-05
glycosaminoglycan binding	242	16	5.25	3.05	+	1.39E-04	4.93E-02
actin binding	451	27	9.79	2.76	+	4.52E-06	3.21E-03
↳cytoskeletal protein binding	1006	45	21.84	2.06	+	8.15E-06	5.07E-03
signaling receptor regulator activity	553	28	12.01	2.33	+	5.75E-05	2.60E-02
calcium ion binding	735	35	15.96	2.19	+	3.01E-05	1.50E-02
Unclassified	2297	16	49.87	.32	-	1.56E-08	1.55E-05
olfactory receptor activity	441	0	9.57	< 0.01	-	1.48E-04	4.91E-02
