# Supplementary material for: Unravelling the Impact of Cyclic Mechanical Stretch in Keratoconus—A Transcriptomic Profiling Study
Source: Int J Mol Sci. 2023 Apr 18;24(8):7437. doi: 10.3390/ijms24087437 (PMC10139219; doi:10.3390/ijms24087437)
Supplement: Supplementary file 1 [file ijms-24-07437-s001.zip › Supplementary Table S5.pdf]

Supplementary Table S5. Cellular component gene ontology terms for 479 DEGs from HKCs (TGFβ1 + CMS) vs. HCFs (TGFβ1 + CMS) (FDR ≤ 0.05).

	Homo sapiens (REF)	Client Text Box Input (▽ Hierarchy NEW! ?)					
GO cellular component complete	#	#	expected	Fold Enrichment	+/-	raw P value	FDR
interstitial matrix	12	4	.26	15.35	+	2.99E-04	2.35E-02
↳collagen-containing extracellular matrix	434	44	9.42	4.67	+	2.86E-16	1.46E-13
↳extracellular matrix	575	57	12.48	4.57	+	2.06E-20	4.21E-17
↳external encapsulating structure	576	57	12.51	4.56	+	2.22E-20	2.27E-17
↳cell periphery	6460	235	140.25	1.68	+	6.44E-20	4.39E-17
↳cellular anatomical entity	18802	432	408.20	1.06	+	1.59E-05	1.92E-03
microfibril	13	4	.28	14.17	+	3.84E-04	2.91E-02
basement membrane	101	13	2.19	5.93	+	9.36E-07	1.74E-04
adherens junction	180	14	3.91	3.58	+	7.34E-05	6.52E-03
↳cell-cell junction	508	29	11.03	2.63	+	4.81E-06	6.55E-04
↳anchoring junction	1336	59	29.01	2.03	+	3.91E-07	7.99E-05
↳cell junction	2136	80	46.37	1.73	+	2.36E-06	4.01E-04
endoplasmic reticulum lumen	314	20	6.82	2.93	+	3.47E-05	3.73E-03
↳cytoplasm	12097	304	262.63	1.16	+	7.93E-05	6.75E-03
actin cytoskeleton	510	24	11.07	2.17	+	6.22E-04	4.38E-02
plasma membrane region	1257	53	27.29	1.94	+	6.42E-06	8.20E-04
↳plasma membrane	5977	191	129.76	1.47	+	1.19E-09	3.47E-07

↳membrane	9956	254	216.15	1.18	+	4.68E-04	3.41E-02
integral component of plasma membrane	1724	71	37.43	1.90	+	3.11E-07	7.07E-05
↳intrinsic component of plasma membrane	1806	74	39.21	1.89	+	2.17E-07	5.55E-05
extracellular exosome	2100	75	45.59	1.65	+	2.72E-05	3.09E-03
↳extracellular vesicle	2123	75	46.09	1.63	+	4.20E-05	4.29E-03
↳extracellular membrane-bounded organelle	2124	75	46.11	1.63	+	4.22E-05	3.92E-03
↳extracellular organelle	2124	75	46.11	1.63	+	4.22E-05	4.10E-03
↳extracellular region	4396	169	95.44	1.77	+	5.04E-15	2.06E-12
↳vesicle	3975	121	86.30	1.40	+	8.19E-05	6.69E-03
↳extracellular space	3430	133	74.47	1.79	+	1.21E-11	4.12E-09
Unclassified	1640	12	35.61	.34	-	4.74E-06	7.45E-04
mitochondrial matrix	486	1	10.55	.09	-	6.31E-04	4.30E-02
