# Supplementary material for: Unravelling the Impact of Cyclic Mechanical Stretch in Keratoconus—A Transcriptomic Profiling Study
Source: Int J Mol Sci. 2023 Apr 18;24(8):7437. doi: 10.3390/ijms24087437 (PMC10139219; doi:10.3390/ijms24087437)
Supplement: Supplementary file 1 [file ijms-24-07437-s001.zip › Supplementary Table S6.pdf]

Supplementary Table S6. PANTHER pathways for 479 DEGs from HKCs (TGFβ1 + CMS) vs. HCFs (TGFβ1 + CMS) (FDR ≤ 0.05).

	Homo sapiens (REF)	Client Text Box Input					
PANTHER Pathways	#	#	expected	Fold Enrichment	+/-	raw P value	FDR
Cadherin signaling pathway	166	19	3.60	5.27	+	1.67E-08	2.67E-06
Integrin signalling pathway	200	16	4.34	3.68	+	1.65E-05	1.32E-03
Wnt signaling pathway	314	20	6.82	2.93	+	3.47E-05	1.85E-03
Unclassified	17971	359	390.16	.92	-	4.21E-05	1.68E-03
