# Supplementary material for: Unravelling the Impact of Cyclic Mechanical Stretch in Keratoconus—A Transcriptomic Profiling Study
Source: Int J Mol Sci. 2023 Apr 18;24(8):7437. doi: 10.3390/ijms24087437 (PMC10139219; doi:10.3390/ijms24087437)
Supplement: Supplementary file 1 [file ijms-24-07437-s001.zip › Supplementary Table S7.pdf]

Supplementary Table S7. Reactome pathways for 479 DEGs from HKCs (TGFβ1 + CMS) vs. HCFs (TGFβ1 + CMS) (FDR ≤ 0.05).

	Homo sapiens (REF)	Client Text Box Input (▽ Hierarchy NEW! ?)					
Reactome pathways	#	#	expected	Fold Enrichment	+/-	raw P value	FDR
Maturation of nucleoprotein	11	4	.24	16.75	+	2.28E-04	4.06E-02
Interferon alpha/beta signaling	68	19	1.48	12.87	+	2.10E-14	5.23E-11
↳Interferon Signaling	197	23	4.28	5.38	+	3.61E-10	4.51E-07
↳Cytokine Signaling in Immune system	697	36	15.13	2.38	+	4.98E-06	2.48E-03
Laminin interactions	30	8	.65	12.28	+	1.09E-06	6.80E-04
Defective B3GALTL causes PpS	38	7	.83	8.48	+	4.21E-05	1.31E-02
O-glycosylation of TSR domain-containing proteins	39	7	.85	8.27	+	4.88E-05	1.35E-02
Integrin cell surface interactions	84	11	1.82	6.03	+	5.54E-06	2.30E-03
Interferon gamma signaling	91	11	1.98	5.57	+	1.11E-05	3.94E-03
Collagen biosynthesis and modifying enzymes	67	8	1.45	5.50	+	1.90E-04	3.64E-02
↳Collagen formation	89	10	1.93	5.18	+	4.90E-05	1.22E-02
Degradation of the extracellular matrix	140	12	3.04	3.95	+	1.02E-04	2.31E-02
Unclassified	10082	179	218.89	.82	-	1.87E-04	3.89E-02
