# Supplementary material for: Unravelling the Impact of Cyclic Mechanical Stretch in Keratoconus—A Transcriptomic Profiling Study
Source: Int J Mol Sci. 2023 Apr 18;24(8):7437. doi: 10.3390/ijms24087437 (PMC10139219; doi:10.3390/ijms24087437)
Supplement: Supplementary file 1 [file ijms-24-07437-s001.zip › Supplementary Table S9.pdf]

Supplementary Table S9. Biological process gene ontology terms for 199 KC-altered genes responsive to TGFβ1 treatment (FDR ≤ 0.05).

	Homo sapiens (REF)	Client Text Box Input (▼ Hierarchy NEW! ?)					
GO biological process complete	#	#	expected	Fold Enrichment	+/-	raw P value	FDR
positive regulation of Wnt signaling pathway, planar cell polarity pathway	9	3	.09	34.84	+	1.73E-04	3.94E-02
↳regulation of signal transduction	2989	53	28.60	1.85	+	6.33E-06	3.82E-03
↳regulation of response to stimulus	4034	67	38.60	1.74	+	2.19E-06	3.12E-03
↳regulation of biological process	11806	143	112.96	1.27	+	1.23E-05	6.42E-03
↳biological regulation	12544	148	120.02	1.23	+	3.52E-05	1.32E-02
↳regulation of cell communication	3369	55	32.24	1.71	+	4.42E-05	1.51E-02
↳regulation of cellular process	11187	133	107.04	1.24	+	2.29E-04	4.67E-02
↳regulation of signaling	3381	56	32.35	1.73	+	2.74E-05	1.16E-02
↳positive regulation of biological process	6304	88	60.32	1.46	+	3.59E-05	1.31E-02
↳regulation of anatomical structure morphogenesis	924	24	8.84	2.71	+	1.17E-05	6.31E-03
↳regulation of developmental process	2489	43	23.82	1.81	+	1.61E-04	3.72E-02
protein mono-ADP-ribosylation	12	4	.11	34.84	+	1.30E-05	6.38E-03
↳protein ADP-ribosylation	29	4	.28	14.42	+	2.58E-04	5.00E-02
fibroblast migration	19	4	.18	22.00	+	6.02E-05	1.93E-02
↳ameboidal-type cell migration	201	9	1.92	4.68	+	1.84E-04	4.06E-02
negative regulation of axon extension involved in axon guidance	27	4	.26	15.48	+	2.02E-04	4.33E-02

↳negative regulation of cellular process	4732	71	45.28	1.57	+	3.94E-05	1.37E-02
↳negative regulation of biological process	5314	81	50.85	1.59	+	3.78E-06	3.12E-03
↳regulation of multicellular organismal development	1383	29	13.23	2.19	+	8.14E-05	2.36E-02
↳regulation of multicellular organismal process	2750	51	26.31	1.94	+	2.79E-06	2.57E-03
↳negative regulation of response to external stimulus	391	13	3.74	3.47	+	1.34E-04	3.22E-02
↳regulation of response to external stimulus	973	23	9.31	2.47	+	7.42E-05	2.20E-02
↳negative regulation of response to stimulus	1620	34	15.50	2.19	+	1.56E-05	7.21E-03
↳regulation of locomotion	1034	23	9.89	2.32	+	2.11E-04	4.41E-02
positive regulation of defense response to virus by host	34	5	.33	15.37	+	3.22E-05	1.23E-02
↳regulation of defense response to virus by host	45	5	.43	11.61	+	1.09E-04	2.85E-02
↳regulation of defense response to virus	76	7	.73	9.63	+	1.39E-05	6.63E-03
↳regulation of defense response	642	19	6.14	3.09	+	1.83E-05	8.18E-03
↳regulation of response to biotic stimulus	364	15	3.48	4.31	+	3.55E-06	3.09E-03
negative regulation of viral genome replication	57	7	.55	12.83	+	2.43E-06	2.54E-03
↳regulation of viral genome replication	87	7	.83	8.41	+	3.15E-05	1.23E-02
↳regulation of viral life cycle	142	8	1.36	5.89	+	9.42E-05	2.50E-02
↳regulation of viral process	164	10	1.57	6.37	+	6.53E-06	3.79E-03
↳negative regulation of viral process	95	10	.91	11.00	+	6.15E-08	2.41E-04
positive regulation of pattern recognition receptor signaling pathway	46	5	.44	11.36	+	1.20E-04	2.98E-02

response to type I interferon	52	5	.50	10.05	+	2.04E-04	4.32E-02
↳response to stimulus	8209	108	78.55	1.37	+	2.78E-05	1.15E-02
↳innate immune response	832	25	7.96	3.14	+	6.09E-07	1.36E-03
↳defense response to other organism	1068	28	10.22	2.74	+	1.70E-06	2.66E-03
↳response to other organism	1429	34	13.67	2.49	+	1.52E-06	2.98E-03
↳biological process involved in interspecies interaction between organisms	1602	34	15.33	2.22	+	1.30E-05	6.56E-03
↳response to external biotic stimulus	1432	34	13.70	2.48	+	1.55E-06	2.71E-03
↳response to biotic stimulus	1478	34	14.14	2.40	+	2.39E-06	2.68E-03
↳response to external stimulus	2469	47	23.62	1.99	+	4.43E-06	3.31E-03
↳defense response	1478	33	14.14	2.33	+	5.90E-06	3.70E-03
↳immune response	1621	35	15.51	2.26	+	7.04E-06	3.94E-03
↳immune system process	2429	47	23.24	2.02	+	2.38E-06	2.87E-03
negative regulation of innate immune response	76	6	.73	8.25	+	1.29E-04	3.17E-02
neural crest cell development	81	6	.78	7.74	+	1.79E-04	4.02E-02
↳stem cell development	87	6	.83	7.21	+	2.58E-04	4.94E-02
↳stem cell differentiation	183	9	1.75	5.14	+	9.35E-05	2.57E-02
↳developmental process	5677	80	54.32	1.47	+	8.24E-05	2.35E-02
↳anatomical structure development	5144	73	49.22	1.48	+	1.87E-04	4.07E-02
↳neural crest cell differentiation	94	7	.90	7.78	+	5.01E-05	1.67E-02
↳mesenchymal cell differentiation	171	9	1.64	5.50	+	5.69E-05	1.86E-02
↳mesenchyme development	239	11	2.29	4.81	+	2.80E-05	1.13E-02

↳tissue development	1726	37	16.51	2.24	+	3.92E-06	3.07E-03
renal tubule development	87	6	.83	7.21	+	2.58E-04	4.88E-02
↳multicellular organism development	4228	66	40.45	1.63	+	2.67E-05	1.16E-02
↳renal system development	308	15	2.95	5.09	+	4.88E-07	1.28E-03
↳urogenital system development	350	15	3.35	4.48	+	2.24E-06	2.93E-03
defense response to virus	253	17	2.42	7.02	+	8.95E-10	7.02E-06
↳response to virus	357	22	3.42	6.44	+	1.29E-11	2.03E-07
↳defense response to symbiont	254	17	2.43	6.99	+	9.47E-10	4.95E-06
kidney development	299	15	2.86	5.24	+	3.41E-07	1.07E-03
extracellular matrix organization	280	13	2.68	4.85	+	4.75E-06	3.39E-03
↳extracellular structure organization	281	13	2.69	4.84	+	4.93E-06	3.36E-03
↳external encapsulating structure organization	283	13	2.71	4.80	+	5.30E-06	3.47E-03
blood vessel development	510	15	4.88	3.07	+	1.53E-04	3.57E-02
↳vasculature development	532	15	5.09	2.95	+	2.38E-04	4.71E-02
embryonic morphogenesis	591	17	5.65	3.01	+	7.19E-05	2.21E-02
↳anatomical structure morphogenesis	2237	39	21.40	1.82	+	2.15E-04	4.44E-02
negative regulation of cell population proliferation	713	19	6.82	2.79	+	7.24E-05	2.18E-02
↳regulation of cell population proliferation	1674	37	16.02	2.31	+	2.49E-06	2.44E-03
cell population proliferation	718	18	6.87	2.62	+	2.35E-04	4.71E-02
regulation of cell migration	929	23	8.89	2.59	+	3.73E-05	1.33E-02
↳regulation of cell motility	989	23	9.46	2.43	+	1.45E-04	3.45E-02

cell adhesion	969	2 3	9.27	2.48	+	6.99E- 05	2.19 E-02
negative regulation of signal transduction	1257	2 6	12.0 3	2.16	+	2.53E- 04	4.95 E-02
positive regulation of multicellular organismal process	1515	3 1	14.5 0	2.14	+	9.02E- 05	2.53 E-02
cellular response to chemical stimulus	2616	4 5	25.0 3	1.80	+	9.36E- 05	2.53 E-02
Unclassified	2725	9	26.0 7	.35	-	1.12E- 04	2.87 E-02
